# Effect of Taichi Softball on Function-Related Outcomes in Older Adults: A Randomized Control Trial

**DOI:** 10.1155/2017/4585424

**Published:** 2017-04-06

**Authors:** Lin Lou, Liye Zou, Qun Fang, Huiru Wang, Yang Liu, Zuguo Tian, Yunpeng Han

**Affiliations:** ^1^Department of Physical Education, North China Electric Power University, Beijing, China; ^2^Department of Physical Education and Health Education, Springfield College, Springfield, MA, USA; ^3^Department of Sports Science, Jishou University, China; ^4^Department of Physical Education, Shanghai Jiao Tong University, Shanghai, China; ^5^Sensorimotor Neurophysiology Laboratory, Indiana University, Bloomington, IN, USA; ^6^College of Physical Education, Hunan University, Hunan, China; ^7^Department of Physical Education, Guiyang Teacher Training College, Guizhou, China

## Abstract

The purpose of this present study was to examine the effect of Taichi softball (TCSB) on physical function in Chinese older adults. Eighty Chinese older adults were randomly assigned into either an experimental group experiencing four 90-minute TCSB sessions weekly for seven consecutive weeks or a control group. At baseline and 7 weeks later, all participants were asked to perform physical functional tests for both lower and upper limbs. Multiple separate Analyses of Variance (ANOVA) with repeated measures were applied to evaluate the effects of TCSB on function-related outcomes between baseline and postintervention in the two groups. The findings indicate that a short-term and intensive TCSB training program does not only improve low limb-related physical function such as dynamic balance and leg strength, but also strengthen upper limb-related physical function (e.g., arm and forearm strength, shoulder mobility, fine motor control, handgrip strength, and fine motor function). Health professionals could take into account TCSB exercise as an alternative method to help maintain or alleviate the inevitable age-related physical function degeneration in healthy older adults. In addition, researchers could investigate the effect of TCSB exercise on physical function in special populations such as patients with different chronic diseases or neurological disorder (e.g., Parkinson's disease).

## 1. Introduction

The total population of adults aged 60 years or older has reached about 123 million in 2013 in China, which accounts for more than 9% of the Chinese population, nearly one in nine citizens. By 2050, this age group is expected to grow to be about 330 million, more than doubling the number in 2013 [1]. People at this age usually experience a decline in sensorimotor control and functioning associated with both lower and upper limbs. The declined motor functions inevitably result in an inferior quality of life in older adults [2].

A great number of studies have been conducted to investigate age-related decline on postural stability, mobility, and hand function. Consistent findings indicate that aging is related to worse performance on postural stability [3–7], mobility [8, 9], and hand function [10–13]. Researchers carried out a cross-sectional, 25-year longitudinal study to investigate whether aging potentially affected strength in upper limbs in 1077 individuals [14]. It was observed that, for both sexes, a decline in power and strength started at the age of 40. More specifically, the decline in power was 10% greater than the decline in strength among men. Similarly, Doherty [15] stated that aging people experienced a decrease in muscle strength, accounting for between 1/5 and 1/2 of the total muscle strength. The loss of muscle strength is associated with a limited mobility which prevents the older adults from good quality of life [16].

Understanding neural mechanism of age-related motor deficits is critical. A degenerative motor cortical region and corpus callosum contribute to an impairment on postural stability and gait [17, 18], coordination [19–21], and movement speed [22]. Declining dopamine as one of the primary neurotransmitter systems was substantiated to have a relationship with age-related gross [23, 24] and fine motor deficits [25, 26]. Greater areas of prefrontal cortex and basal ganglia were involved when older adults performed tasks related to motor control, whereas young adults demonstrated the involvement of fewer areas [27–30].

The fact that an aging population has become larger, which is directly associated with costly geriatric care, stimulates researchers to keep searching for methods to mitigate age-related motor deficits. The ability to prevent brain changes and motor deficits is critical for the older population to efficiently perform daily routines such as cooking, driving, and typing. With an exception of the pharmaceutical method, aerobic exercise interventions hold promise for ameliorating motor deficits associated with normal aging [31–33].

One strategy to mitigate or reverse age-related decline in motor function is to actively participate in physical activity or exercise training. Exercise and other types of physical activities have been recommended in order to gain health benefits in aged individuals. TCSB is a progressive form of health-promoting Taichi exercise that requires practitioners to hold a racket and control a softball on the surface of the racket while performing a Taichi form [34]. A recent study investigating the effect of TCSB exercise on lower limb-related physical function was involved with 32 Chinese older adults with Type 2 diabetes mellitus [35]. However, the effect of TCSB exercise on hand function in the healthy older adults still remains unknown. Therefore, the researcher hypothesized that older individuals practicing TCSB would have better performance in hand function tests when compared to a control group.

## 2. Method

### 2.1. Participants

The study included 80 individuals recruited from a senior living community in the northeast region of China. The individuals were considered eligible for participation in the present study if they (a) were at the age of 55 and above, (b) were free of musculoskeletal injuries or any restricted mobility in both lower and upper limbs, (c) did not currently participate in any other behavioral studies or instructor-led exercise program, (d) are free of vision impairment, and (e) had no severe neurological disease.

### 2.2. Assessments

Primary outcome measures included functional tests for the lower and upper limbs. The outcome measure of the lower limbs involved leg strength and dynamic balance. The outcome measures of the upper limb involved shoulder mobility, handgrip strength, fine motor function, upper body strength and endurance, and fine motor control.

Leg strength was measured with the 30 s Chair Stand Test. Jones et al. [36] investigated the test-retest reliability and the criterion-related validity of the 30-s Chair Stand as a measure of lower body strength in adults over the age of 50 years. The findings of the 30-s Chair Stand Test indicated a test-retest intraclass correlation coefficient of .92 (excellent reliability) and the criterion-related validity of .78. Participants were asked to perform full stands from a seated position while keeping their arms folded across their chests. The number of full stands is recorded, with higher counts indicating better leg strength.

Dynamic balance was measured with the Time Up and Go Test, which was found to have good intrarater and interrater reliability (*r* = .93 and .96, resp.) [37] and validity (*r* = .815) [38]. Participants need to follow the sequential steps for the dynamic balance test: (1) stand up from a seated position; (2) walk 10 feet; (3) make a turn; (4) return to the seated position. The shortest period of time in seconds is recorded. Lower scores indicate better dynamic balance.

The Back Scratch Test was used for measuring shoulder mobility (left and right sides) [39]. The participant was asked to keep a standing position. To perform the Back Scratch Test, the participant needs to (1) place one hand behind the head and over the shoulder as well with his/her palm touching body and the fingers directed downwards while reaching as far as possible down the middle of his/her back; (2) place the other hand behind his/her back, palm facing outward and fingers upward while reaching up as far as possible in order to touch or overlap the middle fingers of both hands. A yardstick was used to measure the distance between the tips of the middle fingers. Shorter distance indicates better flexibility.

The Arm Curl Test was used to measure upper body strength [39]. The frequency of biceps curls in 30 seconds (left and right sides) was recorded. For the Arm Curl Test, the participant needs to perform the following procedures: (1) sit on the chair, holding the dumbbell (e.g., women with 5 lbs, men with 8 lbs) in the hand with palm facing towards the body (with the arm in a vertically down position beside the chair); (2) brace the upper arm against the body so that only the lower arm is moving; (3) curl the arm up through a full range of motion, gradually performing elbow flexion with supination; (4) as the arm is lowered through the full range of motion, gradually return to the starting position. When performing the Arm Curl Test, the participant needs to fully bend and then fully straighten at the elbow. Greater frequencies of the biceps curl indicate stronger strength.

The maximum isometric strength of the hand and forearm muscles was measured using Handgrip Strength Test (criteria validity = .98 and interrater reliability = .996) with a handgrip dynamometer [40, 41]. Participant was asked to use his or her dominant hand to hold the dynamometer in the hand, with the arm at right angles and the elbow by the side of the body, positioning the middle bones of fingers so that participant rests on the forward end of the dynamometer grip. Whenever participant is ready, he or she starts squeezing the dynamometer with maximum isometric effort (lbs.), which is maintained for about five seconds [42]. Each participant was given two trials to carry out the handgrip test; the best performance was recorded.

The Spiral Drawing Test was used for measuring fine movement control. The participant was asked to trace a picture of spiral template on a piece of paper. The participant was instructed to place the pen in the middle of the spiral before tracing starts and then try to trace the spiral as accurately and as fast as possible using their dominant hand and then nondominant hand. The spiral drawing performance was visually evaluated based on the Archimedes Spiral Drawing Test of the International Cooperative Ataxia Rating Scale [43], with a score ranging from 0 to 4. Lower scores indicate better fine motor control.

Moberg Pickup Test (test-retest reliability = .91 and high discriminative validity) was used to measure fine motor function associated with the ability of perceiving constant touch, precision grip, and cutaneous feedback [44]. Participant was asked to pick up small objects used in activities of daily living (e.g., paperclip, safety pin, bolt, nail, washer, wing nut, and small key) from a table and put the objects into a box. Each participant was encouraged to pick up the objects from the table into the box as quickly as possible. Once he or she starts, tester starts timing, with shorter time indicating better fine motor function.

To better understand the effect of TCSB on motor function in older adults, data collection regarding demographic information of participants was performed, including age, gender, marital status, education level, body weight (kg), body height (cm), and monthly income. For obtaining BMI level, body weight and height were assessed through the use of digital scales (Health Meter).

### 2.3. Procedures

Following approval from the Institutional Review Board, the director of a senior living community was contacted to request permission to proceed with the study and recruit participants. Residents in the senior living community were invited to attend an information session about the study. After signing informed consent forms, the eligible individuals were randomly assigned into two groups with an equal number of participants in each group [40]: TCSB and control groups. The present study included three phases: a baseline, 7-week intervention phase, and a postassessment. At the baseline, all individuals were asked to perform functional tests in lower (Chair Stand Test for leg strength, Time Up and Go Test for dynamic balance) and upper limbs (Moberg Pickup Test, Back Scratch Test, Arm Curl Test, Handgrip Strength Test, and Spiral Drawing Test).

During the 7-week intervention phase, participants in the experimental group experienced four TCSB sessions per week for 90 minutes, which were taught by a certified Taichi instructor following the instruction routine: (1) 10-minute instructor-led warm-up, (2) 70-minute well-established exercise form, and (3) 10-minute relaxation. A specific TCSB exercise was developed by a Taichi certified instructor in order to improve motor function in both lower and upper limbs. Participants in the control group were asked to keep their original lifestyles during the intervention period.

**Figure 1:**
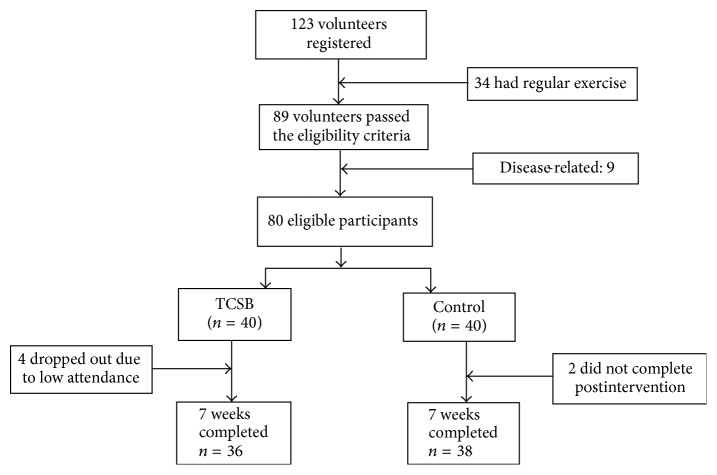
Screening, randomization, and completion of 7 weeks.

At the end of the 7-week intervention, a postassessment with similar testing procedures to those of the baseline was administrated to all participants. All tests were conducted at the indoor fitness room of the community centers. Each participant had two trials for each functional test which was administered by an experienced researcher. A 3-minute break between the two tests was implemented to ensure adequate recovery. The best result of the two trials was used for data analysis (Figure 1).

## 3. Statistical Analyses

All statistical analyses were carried out with IBM SPSS version 23.0. The level of significance for the present study was set at .05. Prior to the beginning of examining the main interest of the present study, preliminary analyses were conducted to examine the demographic variables (e.g., age, gender, and education level). Means and standard deviations were used to summarize continuous data and frequency was used to summarize categorical data. Differences at baseline for the demographic information between the two groups were compared using a *t*-test for continuous data and a Chi-squared test for categorical data.

To evaluate the within-group effects of outcome measures between pre- and postintervention, multiple separate pair *t*-tests for each group were used in the present study, followed by multiple separate Analyses of Variance (ANOVA) with repeated measures to examine whether interaction effect of group and time for each outcome existed.

## 4. Results

### 4.1. Demographic Information of Participants

The demographic information of participants in both TCSB and control groups is presented in Table 1. The mean ages were 62.2 (SD = 3.43) in the TCSB group and 62.6 (SD = 3.54) in the control group, respectively. Female participants accounted for 45% (*n* = 18) in the TCSB and 40% (*n* = 16) in the control group, respectively. The majority of participants in both groups graduated with high school diploma (TCSB = 47.5%; control group = 42.5%). 37.5% of participants in the TCSB group fell under the category of normal BMI when 45% of participants in the control group belong to normal BMI range, followed by 32.5% versus 25% (25–25.9), 15% versus 17.5% (less than 18.5), 15% versus 12.5% (30–34.9), and 0% versus 0% (35–39.9). Marital status of participants was classified into single, married, and divorced/separated/widowed in the TCSB (single = 25%, married = 55%, and divorced/separated/widowed = 20%) and control group (single = 30%, married = 57.5%, and divorced/separated/widowed = 12.5%). For the monthly income perspective, participants of both groups fell under the range from 2,500 to 4,999 (TCSB = 57.5%, control  group = 65%).

### 4.2. Effects of Intervention

Table 2 demonstrates the within-group and between-group differences of motor function-related outcomes for the two groups, including fine motor control, fine motor function, handgrip strength, arm strength and endurance, shoulder mobility, leg strength, and dynamic balance as measured by Spiral Drawing Test, Moberg Pickup Test, Handgrip Strength Test, Arm Curl-Up Test, Back Scratch Test, Chair Stand Test, and Timed Up and Go Test, respectively. At baseline, no significant difference was observed on all the outcome measures between the TCSB and control groups (*p* > 0.05). At week 7, the TCSB group had significant improvements in all the outcome measures (*p* < 0.0001 for all outcome measures), in comparison to the control group experiencing deterioration of these outcome measures. More specifically, the TCSB group experienced positive changes on Spiral Drawing Test (.67 points, 3.11 to 2.44), Moberg Pickup Test (3.22 points, 21.61 to 18.39), Handgrip Strength Test (−4.08, 16.50 to 20.58), Arm Curl-Up Test (−4.03 points, 18.31 to 22.33), Back Scratch Test (3.63 points, 14.57 to 10.93), Chair Stand Test (−6.75 points, 13.47 to 20.22), and Timed Up and Go Test (3.58 points, 14.74 to 11.16).

The between-group difference in the change of each outcome measure was then examined by interaction effect of time and group. When compared to the control group, fine motor control [*F*(1, 72) = 29.58, *p* < 0.0001], fine motor function [*F*(1, 72) = 44.76, *p* < 0.0001], handgrip strength [*F*(1, 72) = 39.26, *p* < 0.0001], hand and forearm strength [*F*(1, 72) = 34.09, *p* < 0.0001], shoulder mobility [*F*(1, 72) = 124.58, *p* < 0.0001], leg strength [*F*(1, 72) = 117. 09, *p* < 0.0001], and dynamic balance [*F*(1, 72) = 213.15, *p* < 0.0001] were significantly improved in the TCSB group. No adverse effects were reported in both groups during the intervention period.

## 5. Discussion

This randomized, controlled study was designed to examine the effect of a 7-week TCSB training program on motor function in Chinese healthy older adults. The findings of this present study demonstrated that a short-term and intensive TCSB training program could improve not only low limb-related motor function such as dynamic balance and leg strength, but also upper limb-related motor function (e.g., arm and forearm strength, shoulder mobility, fine motor control, handgrip strength, and fine motor function).

The number of individuals over 60 years is exponentially growing and expected to grow from 123 million in 2013 to roughly 330 million by 2050 in China [1]. Given that biological aging was generally associated with deterioration and the deprivation of functional health [45–47], one of the most common and serious public health problems caused by this age-related degeneration is that older adults are experiencing an increase in the chance of falling. Therefore, measuring lower limb strength and balance is critical in evaluating the functional performance of older adults because these physical capabilities play a significant role in carrying out a variety of tasks such as climbing stairs, walking and getting out of a chair, tub, or car, or avoiding fall. The present study included two lower body outcome measures with regard to leg strength and dynamic balance, as measured by the Chair Stand Test and Timed Up and Go Test, respectively. TCSB group was able to perform around seven more transition movements (13.47 to 20.22) from sitting to standing position after the 7-week intervention, indicating that participants had obtained an improvement on leg strength through twenty-eight 90-minute TCSB training sessions. This encouraging finding coincided with a previous study examining the effectiveness of TCSB on leg strength as measured by the same Chair Stand Test in Chinese older adults from Hong Kong special administrative region [34]. With the increased muscular strength in low limbs, it is reasonable to observe an improvement in the dynamic balance performance in the present study as well. It may be because the increased leg strength building a stronger base of support allows older adults to use wider stride, leading to a faster walking speed so that they can complete Timed Up and Go Test in a shorter period of time in comparison to the baseline condition [34, 35]. Participants in the TCSB group experienced both leg strength and dynamic balance improvement, which is possibly attributed to the fact that TCSB is characterized by constant weight shifting movements from left to right side of body or change in the base of support from double- to single-leg standing. Unfortunately, participants in the control group experienced deterioration of all the outcome measures in the present study, which may be due to the biological aging [48, 49].

Because TCSB was developed according to the principle of Taichi movements, the positive effects on lower limb outcome measures were observed in the present study that provides additional evidence to support the beneficial effects of Taichi-based exercises on leg strength and physical balance for fall prevention purpose [50]. However, existing literature regarding the effect of Taichi-based exercises on upper limb outcome measures is very limited. In fact, upper limb functional capabilities are also critical in order for older adults to efficiently and smoothly perform activities of daily living. For example, upper limb manipulative skills are needed to use a telephone, hold a cooking utensil, and insert a key into the key hole [51]. Hoogendam et al. [12] recruited a relatively large sample size (*n* = 1,912 community-dwelling middle-aged and older adults) to determine older age contributing to worse upper limb manipulative skills [12, 52]. Therefore, the present study contained a relatively comprehensive upper limb testing instrument (fine movement control, fine manipulative skill, handgrip strength, hand and forearm strength and endurance, and shoulder mobility) to investigate whether TCSB was effective in alleviating or maintaining age-related upper limb functional capability degeneration. The findings of the present study are encouraging because in comparison to participants in the control group suffering from normal aging-related degeneration in the upper limb, participants in the TCSB group demonstrated significantly greater improvement on the five outcome measures.

Based on the encouraging findings, researchers of the present study encourage future studies to compare TCSB with Taichi form for older adults. If TCSB was as effective as Taichi in improving lower limb-related functional capabilities such as leg strength and physical balance, TCSB had more positive effects on upper limb manipulative skill performance than Taichi. In this way, health professionals could establish a more well-rounded Taichi-based exercise program according to the progressive principle [53, 54]. In addition, TCSB may be beneficial for patients with Parkinson's disease to alleviate their symptoms, particularly for resting tremor [55–57]. No adverse effect was reported in the present study, indicating that TCSB is a safe form of exercise. In addition, TCSB as a mind-body integrative exercise is slightly different from traditional Taichi forms because when compared to most of the traditional Taichi forms belonging to individual-based exercise, TCSB can be practiced with peers, which is more enjoyable. A substantial number of studies indicated that social isolation was highly associated with psychiatric morbidity and mortality risks [58–60]. A recently published study indicated that peer-assisted Qigong exercise was effective in strengthening social interaction and improving psychological well-being in Chinese hidden elders, older adults who are socially isolated and refuse social participation [58]. Thus, TCSB as a peer-based enjoyable exercise may be suitable to be considered as a feasible social intervention for this special population.

As we know the control group maintained their normal lifestyles during the intervention period; however, within-group analysis showed that the control group had significant differences in assessment tests such as Spiral Drawing Test, Moberg Pickup Test, Arm Curl-Up Test, Back Scratch Test, Chair Stand Test, and Time Up and Go Test between baseline and after 7 weeks (Table 2). Participants from both groups were recruited from a senior living center; previous studies had shown that, without exercise, motor functions of older people would experience an age-related normal decline, which corresponds to our findings [34, 61]. For timing tests such as Moberg Pickup Test and Time Up and Go Test, we see a significant decrease in fine motor perception as well as a decrease in whole body movement speed. The Spinal Drawing Test for the control group increased through time; a higher score represents worse motor control, which means, without exercise intervention, seniors could not even maintain their movement ability. The Arm Curl-Up Test showed arm curling frequency in the control group also decreased through time, which means that the controls were losing upper arm muscle strength. The Back Scratch Test showed, unlike TCSB group, the control group increased the distance between the two hands without participating in any exercises. The Chair Stand Test is an indicator of leg strength; the control group showed a significant decrease in leg strength after the intervention. Compared to the significant decrease in above tests in control subjects, we can see that, without exercise, seniors are losing strength and mobility; while practicing TCSB, instead of losing motor control or maintaining the control, participants can instead reverse the declining trend and improve their movements.

Although the findings of the present study are promising, some limitations of this study should be mentioned. First of all, due to the present study involving a short-term and intensive TCSB training, whether the lasting positive effects of TCSB on health-related outcomes exist remains unclear. Therefore, a long-term TCSB intervention with a follow-up assessment should be designed. Second, eating habit or diet may affect outcomes, which should be taken into consideration and adjusted in future studies. For the future investigation, the TCSB should be compared to other forms of exercises such as walking, to see if TCSB could be a better overall intervention exercise by not only slowing down the declining trend of movements but also improving.

## 6. Conclusion

The findings of the present study indicate that TCSB exercise is potentially effective in strengthening the physical functional health, including leg strength, dynamic balance, mobility, fine motor function and control, hand and forearm muscular strength, handgrip strength, and shoulder mobility, thereby ultimately enhancing the quality of life in older adults. Health professionals could take into account TCSB exercise as an alternative method to help maintain or alleviate the inevitable age-related physical function degeneration in healthy older adults. In addition, researchers could investigate the effects of TCSB function-related outcomes in special populations such as patients with different chronic diseases or Parkinson's disease.

## Figures and Tables

**Table 1:** Demographic information of participants at baseline.

Demographic	TCSB (*n* = 40)		Control (*n* = 40)		*p*
	Mean (SD)	*N* (%)	Mean (SD)	*N* (%)	
Age	62.2 (3.43)		62.6 (3.54)		>0.05

Gender					>0.05
Male		22 (55%)		24 (60%)	
Female		18 (45%)		16 (40%)	

BMI					>0.05
Less than 18.5		6 (15%)		7 (17.5%)	
18.5–24.9 (normal)		15 (37.5%)		18 (45%)	
25–25.9		13 (32.5%)		10 (25%)	
30–34.9		6 (15%)		5 (12.5%)	
35–39.9		0 (0%)		0 (0%)	

Educational level					>0.05
High school diploma		19 (47.5%)		17 (42.5%)	
Associate's degree		7 (17.5%)		7 (17.5%)	
Bachelor's degree		9 (22.5%)		11 (27.5%)	
Master's degree or above		5 (12.5%)		5 (12.5%)	

Marital status					>0.05
Single		10 (25%)		12 (30%)	
Married		22 (55%)		23 (57.5%)	
Divorced/separated/widowed		8 (20%)		5 (12.5%)	

Monthly income					0.783
<2,500		8 (20%)		5 (12.5%)	
2,500–4,999		23 (57.5%)		26 (65%)	
5,000–9,999		6 (15%)		5 (12.5%)	
≥10,000		3 (7.5%)		4 (10%)	

**Table 2:** Within-group and between-group comparisons for outcome measures at baseline and week 7 (*n* = 74) using repeated measures ANOVA.

Measure	Within-group effects			Between-group effects		
	Baseline	Week 7	*p*	Baseline–Week 7	Time by group	
	Mean (SD)	Mean (SD)		Mean (SD)	*F*(1, 72)	*p*
Spiral Drawing Test					29.58	<0.0001
Taichi softball (*n* = 36)	3.11 (.94)	2.44 (.77)	<0.0001	.67 (.76)		
Control group (*n* = 38)	2.18 (.87)	3.18 (.87)	0.002	−.37 (.67)		

Moberg Pickup Test					44.76	<0.0001
Taichi softball (*n* = 36)	21.61 (8.35)	18.39 (6.71)	<0.0001	3.22 (3.33)		
Control group (*n* = 38)	21.11 (8.09)	21.95 (8.30)	0.004	−.84 (1.67)		

Handgrip Strength Test					39.26	<0.0001
Taichi softball (*n* = 36)	16.50 (4.00)	20.58 (4.20)	<0.0001	−4.08 (4.17)		
Control group (*n* = 38)	16.63 (4.02)	16.34 (3.71)	0.086	.290 (1.01)		

Arm Curl-Up test					34.09	<0.0001
Taichi softball (*n* = 36)	18.31 (5.99)	22.33 (4.97)	<0.0001	−4.03 (4.42)		
Control group (*n* = 38)	17.89 (5.23)	17.71 (5.10)	0.033	.18 (.51)		

Back Scratch Test					124.58	<0.0001
Taichi softball (*n* = 36)	14.57 (4.76)	10.93 (5.05)	<0.0001	3.63 (2.04)		
Control group (*n* = 38)	14.51 (4.39)	15.40 (4.69)	<0.0001	−.89 (2.23)		

Chair Stand Test					117.09	<0.0001
Taichi softball (*n* = 36)	13.47 (2.43)	20.22 (3.32)	<0.0001	−6.75 (3.70)		
Control group (*n* = 38)	14.02 (2.61)	13.13 (1.89)	0.019	.89 (2.24)		

Timed Up and Go Test					213.15	<0.0001
Taichi softball (*n* = 36)	14.74 (1.22)	11.16 (.65)	<0.0001	3.58 (1.23)		
Control group (*n* = 38)	14.25 (1.00)	15.27 (1.61)	<0.0001	−1.03 (1.47)		
