# Corrigendum to “Effect of Taichi Softball on Function-Related Outcomes in Older Adults: A Randomized Control Trial”

**DOI:** 10.1155/2017/2186987

**Published:** 2017-11-02

**Authors:** Lin Luo, Liye Zou, Qun Fang, Huiru Wang, Yang Liu, Zuguo Tian, Yunpeng Han

**Affiliations:** ^1^Department of Physical Education, North China Electric Power University, Beijing, China; ^2^Department of Physical Education and Health Education, Springfield College, Springfield, MA, USA; ^3^Department of Sports Science, Jishou University, China; ^4^Department of Physical Education, Shanghai Jiao Tong University, Shanghai, China; ^5^Sensorimotor Neurophysiology Laboratory, Indiana University, Bloomington, IN, USA; ^6^College of Physical Education, Hunan University, Hunan, China; ^7^Department of Physical Education, Guiyang Teacher Training College, Guizhou, China

In the article titled “Effect of Taichi Softball on Function-Related Outcomes in Older Adults: A Randomized Control Trial” [1], the name of the first author was given incorrectly as Lin Lou. The author's name should have been written as Lin Luo. The revised authors' list is shown above.
